# High-level production of pullulan and its biosynthesis regulation in *Aureobasidium pullulans* BL06

**DOI:** 10.3389/fbioe.2023.1131875

**Published:** 2023-01-26

**Authors:** Shuyu Chen, Hongchen Zheng, Jiaqi Gao, Hui Song, Wenqin Bai

**Affiliations:** ^1^ Colleg of Biotechnology, Tianjin University of Science Technology, Tianjin, China; ^2^ CAS Key Laboratory of Systems Microbial Biotechnology, Tianjin Institute of Industrial Biotechnology, Chinese Academy of Sciences, Tianjin, China; ^3^ National Center of Technology Innovation for Synthetic Biology, Tianjin, China; ^4^ Industrial Enzymes National Engineering Research Center, Tianjin Institute of Industrial Biotechnology, Chinese Academy of Sciences, Tianjin, China; ^5^ Key Laboratory of Engineering Biology for Low-carbon Manufacturing, Tianjin Institute of Industrial Biotechnology, Chinese Academy of Sciences, Tianjin, China

**Keywords:** pullulan, aureobasidium pullulans, biosynthesis, high-level production, high molecular weight, moderate molecular weight, food preservation

## Abstract

Pullulan has many potential applications in the food, pharmaceutical, cosmetic and environmental industries. However, the yield and molecular properties of pullulan produced by various strains still need to be promoted to fit the application needs. A novel yeast-like strain *Aureobasidium pullulans* BL06 producing high molecular weight (Mw) pullulan (3.3 × 10^6^ Da) was isolated and identified in this study. The remarkable Mw of pullulan produced by *A. pullulans* BL06 was the highest level ever reported thus far. To further regulate the biosynthesis of pullulan in *A. pullulans* BL06, three gene knockout strains *A. pullulans* BL06 ΔPMAs, *A. pullulans* BL06 Δmel, and *A. pullulans* BL06 ΔPMAsΔmel, were constructed. The results showed that *A. pullulans* BL06 ΔPMAs could produce 140.2 g/L of moderate Mw (1.3 × 10^5^ Da) pullulan after 120 h of fermentation. The highest yield level of pullulan to date could vastly reduce its production cost and expand its application scope and potential. The application experiments in food preservation showed that the moderate-Mw pullulan obtained in this work could reduce the weight loss of celery cabbages and mangos by 12.5% and 22%, respectively. Thus, the novel strains *A. pullulans* BL06 and *A. pullulans* BL06 ΔPMAs possessed unlimited development prospects in pullulan production at various Mw ranges and pullulan applications in multiple fields.

## 1 Introduction

Pullulan, composed of repeating maltotriose units connected by α-(1, 6) glycosidic bonds, is a linear extracellular polysaccharide (EPS) that is usually produced by the yeast-like fungus *Aureobasidium pullulans* (*A. pullulans*) (Singh et al., 2021; Wei et al., 2021). It exhibits a wide range of applications in biomedicine, environmental engineering, and food engineering because it is a renewable, toxin-free, non-immunogenic and non-carcinogenic natural polymer (Singh et al., 2019; Nishimura et al., 2020; An et al., 2021; Feng et al., 2022). More specifically, the unique linkage pattern ofα-D-glucan endows pullulan with excellent solubility, film-forming ability, oxygen barrier ability, and structural elasticity to meet the requirements for application in drug delivery, gene targeting, tissue engineering, wound healing, and 3D/4D printing (Duan et al., 2020; Ghorbani et al., 2020; Moraes et al., 2020; Shah et al., 2021; Feng et al., 2022). The intramolecular and intermolecular polyhydroxyl interactions of the linear pullulan molecule are key to its superb material properties. High-molecular-weight (High-Mw) pullulan could offer stronger mechanical properties due to its longer polysaccharide chain and its richer hydroxyl content, which makes it a promising candidate in biomedical materials and shape memory polymers (SMPs) (Jiang et al., 2019; Feng et al., 2022). However, pullulan-based hard capsules were recently manufactured by using commercial pullulan (e.g., Hayashibara Co., Ltd., Japan) with a mean molecular weight (Mw) of 1.0 × 10^5^ or 2.0 × 10^5^ Da (Liu et al., 2018; Ding et al., 2020). Besides, to date, the application of pullulan in various fields has been actually limited due to both the low production yield and low Mw of pullulan (Liu et al., 2018). Owing to the importance of the properties and production cost of pullulan, bioprocessing and strain modification have been widely studied to enhance the Mw and yield of pullulan. Additionally, efficient genome editing techniques which were established in 2019 also provide the assistance to regulate the relevant enzymes and genes of the pullulan biosynthesis pathway in different strains of *Aureobasidium* spp. (Zhang et al., 2019). For example, simultaneous removal of both duplicated AMY1 genes encoding α-amylase and duplicated PKS1 genes responsible for melanin biosynthesis in *A. melanogenum* TN3-1 rendered a mutant AMY-PKS-11 to transform 140.0 g/L glucose to produce 103.50 g/L pigment-free pullulan with a Mw of 3.2 × 10^5^ Da (Chen et al., 2019; Xue et al., 2019; Chen et al., 2020). A triple mutant DT15 grown at the flask level could produce 46.2 g/L of pullulan with a Mw of 3.02 × 10^6^ Da and grown in a 10-L fermentor could yield 58.14 g/L pullulan with the same Mw, while its wild-type strain P16 produced 65.5 ± 3.5 g/L pullulan with a Mw of 0.35 × 10^6^ Da (Liu et al., 2018). Thus, more efforts should be made to enhance pullulan production and improve the chemical properties of pullulan *via* molecular modifications of the producers by using synthetic biology approaches.

Pullulan produced by *A. pullulans* fermentation normally contains byproducts, including melanin, glucan and polymalic acid (Zeng et al., 2020; Chen et al., 2021; Liu et al., 2021). The presence of these impurities largely increases the difficulty of pullulan purification. Due to differences in metabolic pathways and cell morphology, the biosynthetic mechanisms vary by different strains and therefore cause the varied product molecular weight among different strains (Sugumaran and Ponnusami, 2017). In this work, the high yield pullulan producing strain *A. pullulans* BL06 was screened from the environment. Pullulan synthesized by this strain showed a molecular weight of 3.3 × 10^6^ Da and a fermentation yield of 83.4 g/L in a 5 L bioreactor. The Mw of the novel identified pullulan is the highest among those of the pullulan ever reported. The strain BL06 combines the advantages of both high molecular weight and high yield and therefore has a high potential for commercialization. Furthermore, by knocking out the polymalic acid (PMA) synthase gene, we obtained another industrial strain *A. pullulans* BL06 ΔPMAs that are capable of producing high purity and moderate Mw pullulan with a high yield. The fermentation of the modified strain reached 140.2 g/L yield of 1.3 × 10^5^ Da pullulan in a 5 L bioreactor, free of melanin and PMA impurities. The application experiments in food preservation of the moderate Mw pullulan obtained in this work were also performed to evaluate its developing potential in the food industry.

## 2 Materials and methods

### 2.1 Strains, plasmids, and chemicals

All of the wild strains producing exopolysaccharides in this study were isolated from the fallen leaves of the park (N39°8′E117°23′) near the Tianjin institute of industrial biotechnology, Chinese academy of sciences in September 2020. All the plasmids used in this work are listed in Supplementary Table S1. *E. coli* DH5α was used to preserve and amplify the recombinant shuttle plasmids. Maltotriose standard, pullulan standard and pullulanase were purchased from Sigma (St. Louis, MO, United States). All other chemicals were of analytical reagent grade purity and obtained from commercial sources.

### 2.2 Screening of the pullulan-producing strains

**FIGURE 1:**
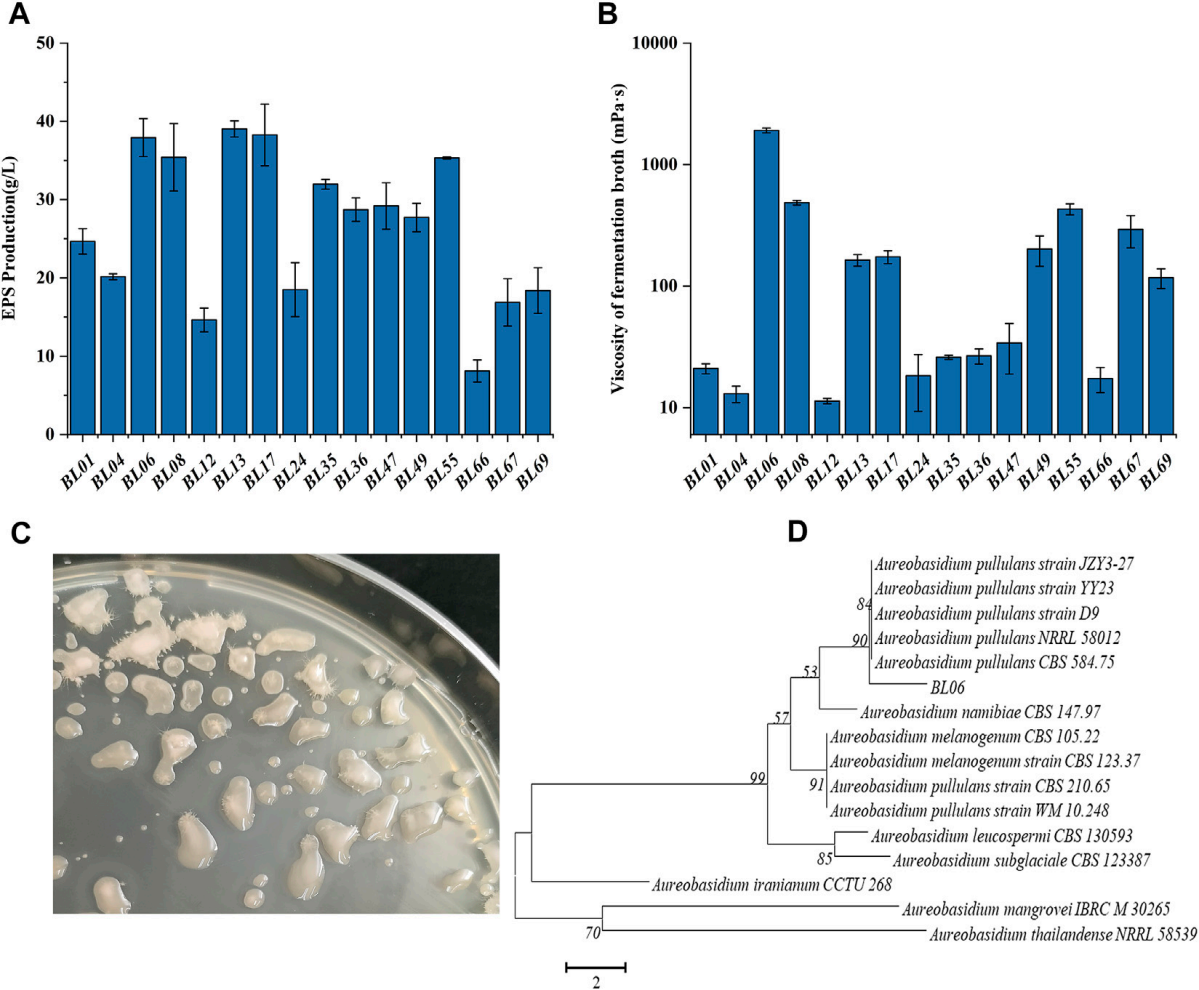
Screening and identification of wild strains for high production of EPS. **(A)** EPS production of the selected wild strains; **(B)** Viscosity of the fermentation broth of the selected wild strains; **(C)** Colonial morphology of strain BL06 on a PDA plate for 120 h of cultivation; **(D)** Phylogenetic tree of strain BL06 and other yeast-like fungal species relatives based on a neighbor-joining analysis of ITS sequences. All values are expressed as the means ± SDs (*n* = 3).

According to previous reports, most of the pullulan producers were isolated from various plant leaves and flowers (Ma et al., 2014). Four kinds of fallen leaves from different trees were used as the sources for fungus isolation in this study. The detailed location information was described in Section 2.1. The fungal-carrying sample obtained from the environment was washed and serially diluted with 0.9% sodium chloride solution and then spread on a potato dextrose agar (PDA) plate, which was 100 mL of potato extract containing 12.0 g of sucrose and 2.0 g of agar with a certain amount of bacterial antibiotics (ampicillin 140 μg/mL, chloromycetin 200 μg/mL), followed by incubation at 28°C for 24 h. After incubation and colony formation, single colonies which without pigment generation and showed smooth and moist in the surface were chosen to identify the production ability of EPS. The yeast-like properties of the single colonies were referred to the standard colonial morphology as shown in Figure 1C. The yeast-like fungal single colonies were further aerobically cultivated in YPD medium (consisting of 1.0% yeast extract, 2.0% polypeptone and 2.0% glucose) at 28°C for 24 h to obtain seed culture. A total of 5 mL of the culture was inoculated into a 250 mL flask containing 50 mL of the pullulan producing media and then incubated at 28°C and 200 rpm for 7 days. After that, the fermentation broth was centrifuged at 14,000 × g for 30 min to remove the fungus cells. Then, 30 mL of the supernatant was taken to detect viscosity using a viscosimeter (DV3T, BROOKFIFLD, United States) with a 61-64^#^ rotor at 30 rpm for 5 min. Collection and quantification of pullulan were performed according to the protocol described below. The pullulan producing media contained 140.0 g/L sucrose, 3.0 g/L yeast extract, 5.0 g/L K_2_HPO_4_, 0.2 g/L MgSO_4_·7H_2_O, 0.01 g/L NaCl.

### 2.3 Phenotypic and molecular analysis of the fungal strain

The phenotypic analysis of the colonies formed on the PDA plates was performed in accordance with the methods described previously (Jiang et al., 2018; Liu et al., 2020). The genomic DNA of strain BL06 was extracted using a TIANamp Yeast DNA Kit (TIANGEN, Beijing, China). Amplification and sequencing of the internal transcribed spacer region (ITS) of the rRNA gene cluster were performed using the common primers ITS1 and ITS4 (Supplementary Table S2) according to the methods described by Ma et al. (2014). The obtained ITS sequence of strain BL06 was aligned using BLAST analysis (http://blast.ncbi.nlm.nih.gov/Blast.cgi). The phylogenetic tree was made in MEGA7.0 by the neighbor-joining method (Kumar et al., 2016; Liu et al., 2020).

### 2.4 Purification and quantification of pullulan

**FIGURE 2:**
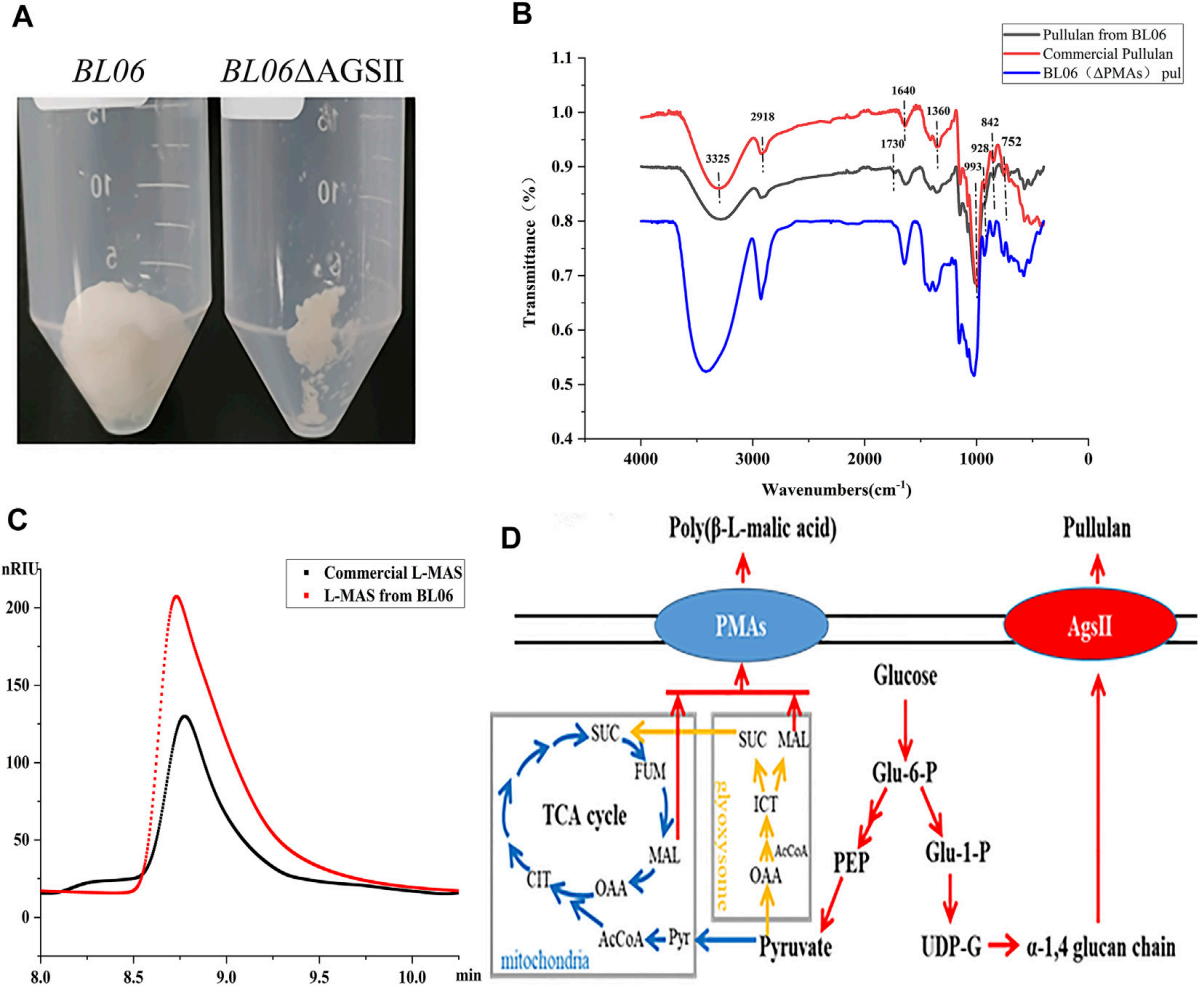
Identification and properties of the EPS of *A. pullulans* BL06. **(A)** The purified EPS produced by *A. pullulans* BL06 and *A. pullulans* BL06ΔAGSII; **(B)** FTIR spectra of the purified EPS and standard pullulan; **(C)** HPLC analysis of the hydrolysate of the purified EPS produced by *A. pullulans* BL06ΔAGSII and L-malic acid standard. **(D)** Synthetic pathways of pullulan and polymalic acid in *A. pullulans*.

The purification and quantification of pullulan were performed according to the methods reported by Ma et al. (2014) with slight modification. The fermentation broth was centrifuged at 4°C 14,000 × g for 10 min to remove the cell pellet. After that, twice the volume of ice-cold ethanol was added to the supernatant. The mixture was then incubated at 4°C for 24 h to precipitate pullulan. The precipitate was dissolved in deionized water at 80°C, followed by a repetitive ethanol precipitation procedure. The collected precipitates were lyophilized to quantify the dry weight of the purified pullulan from the fermentation products (Figure 2A).

### 2.5 Characterization of pullulan

To characterize the purified pullulan, high performance liquid chromatography (HPLC), fourier-transform infrared spectrometer (FT-IR) analysis (Jiang et al., 2019), right angle light scattering (RALS) (Liu et al., 2018), and high performance gel permeation chromatography-multiangle laser light scattering (HPGFC-MALLS) (Thomsen, 2020) were performed according to previous reports. The commercial pullulan (viscosity 100–180 mPa·s) and the experimental pullulan were dissolved in ddH_2_O to obtain a 10 mg/mL pullulan solution, respectively. Taken 1 mL of the pullulan solution to be hydrolyzed by 2 U/mL pullulanase in 50 mM sodium acetate-acetic acid buffer (pH 4.5) at 60°C for 30 min. Then, the mixture was boiled for 10 min to terminate the hydrolysis reaction. The samples were then centrifuged at 14,000 × g for 10 min to collect the supernatant. The supernatant of hydrolysates was filtrated with 0.22 μm syringe filters before HPLC analysis. An Agilent 1260 HPLC with a refractive index detector (RID) and a Bio-Rad Aminex HPX-87P column was used in the analysis. The mobile phase was ultra-pure water with a flow rate of 0.6 mL/min at 65°C. In addition, maltotriose was used as an external standard for analysis of the hydrolysates. The lyophilized purified pullulan (4 mg) obtained in Section 2.4 was mixed with 120 mg of 95% potassium bromide powder and then desiccated overnight at 50°C under vacuum. FT-IR spectra were measured and recorded using potassium bromide pellets of the purified pullulan and the pullulan standard over an arrangement of 4,000–400 cm^−1^ at a rate of 16 scans with a resolution of 2 cm^−2^ using FT-IR (a Nicolet Nexus FTIR 470 spectrophotometer) (Jiang et al., 2019)*.* The Mw of the purified pullulan was determined using RALS and HPGFC-MALLS, respectively. The lyophilized purified pullulan was dissolved in 0.1 N sodium nitrate solution. The sample was measured using RALS (Viscotek TDA305max, Malvern, UK) with an AGuard +1 × A6000 column. The mobile phase was 0.1 N sodium nitrate solution with a flow rate of 0.8 mL/min at 30°C (Liu et al., 2018). HPGFC-MALLS was specially used to determine the high Mw pullulan sample with a Mw above 3.0 × 10^6^ Da. The lyophilized purified pullulan was dissolved in 0.1 N sodium nitrate solution to a final concentration of 5 mg/mL. The sample was measured using an HPLC (Agilent 1260) with a wyatt multiangle light scattering detector and a Shodex OHpak SB-805 HQ column. The mobile phase was 0.1 N sodium nitrate solution with a flow rate of 0.5 mL/min at 40°C (Thomsen, 2020).

### 2.6 Gene knockout of *A. pullulans* BL06

**FIGURE 3:**
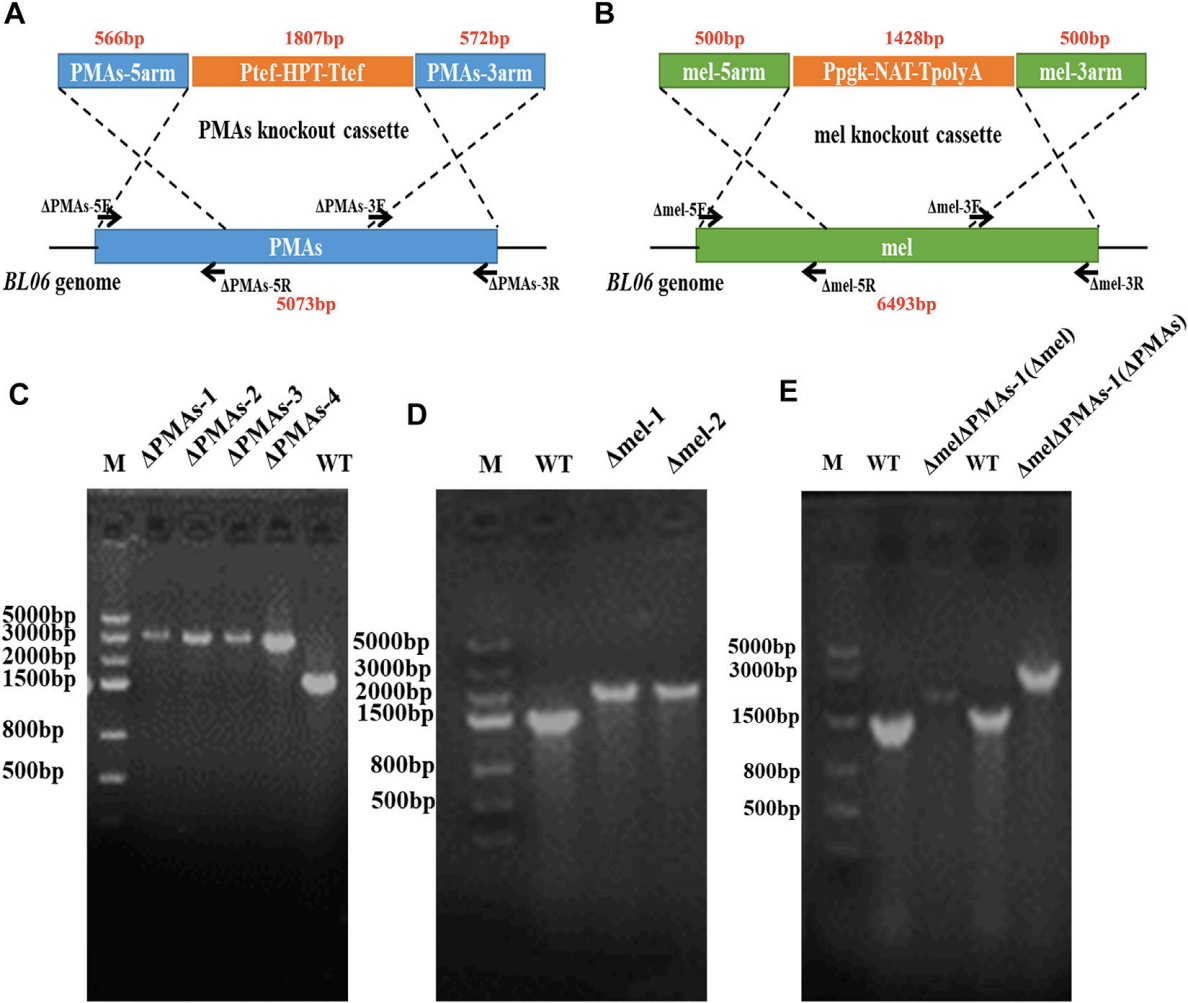
Construction of engineered strains for gene knockout. **(A)** Schematic diagram of gene knockout of PMAs in the genome of *A. pullulans* BL06; **(B)** Schematic diagram of gene knockout of melanin in the genome of *A. pullulans* BL06; **(C)** Identification of the positive transformants for *A. pullulans* BL06ΔPMAs by PCR; **(D)** Identification of the positive transformants for *A. pullulans* BL06Δmel by PCR; **(E)** Identification of the positive transformants for *A. pullulans* BL06ΔPMAsΔmel by PCR.

The primers ΔAGSII-5F/5R, ΔAGSII-3F/3R, Δmel-5F/5R, Δmel-3F/3R, ΔPMAs-5F/5R, and ΔPMAs-3F/3R (Supplementary Table S2), used to amplify the homologous arm sequences of the pullulan synthase (AGSII) gene, polyketide synthase (PKS) gene, and polymalate synthase (PMAs) gene, were designed according to the genome sequences of *Aureobasidium melanogenum* P16, *Aureobasidium pullulans* As3.3984, and *Aureobasidium melanogenum* ATCC62921, respectively. The genome integration plasmids pSY018, pSY005, and pJQ046 (Supplementary Table S1 and Supplementary Figure S1) depending on homologous recombination were constructed by assembling the gene replacement boxes AGSII-5′arm-P*pgk*-NAT-T*polyA*-AGSII-3′arm, mel-5′arm-P*pgk*-NAT-T*polyA*-mel-3′arm, and PMAs-5′arm-P*tef*-HPT-T*tef*-PMAs-3′arm (Figure 3A,B) using the golden gate method. *Nat* and *Hpt*, the codon-optimized nourseothricin resistance gene and hygromycin B resistance gene, were expressed under the promoters of P*pgk* and P*tef* in the gene expression boxes, respectively (Figure 3A,B). The linear DNA was amplified by polymerase chain reaction (PCR) using ΔAGSII-5F/3R, Δmel-5F/5R, and ΔPMAs-5F/3R as primers from the templates of pSY018, pSY005, and pJQ046, respectively. The amplified DNAs (1–2 μg) were transformed into protoplasts of *A. pullulans* BL06 through the electro-transformation method. The recombinant strains were screened on YPD plates containing NAT or HPT at a concentration of 50.0 μg/mL. The positive engineered strains were determined by PCR and sequencing of the gene integration site on the genome. The double-gene knockout strain *A. pullulans* BL06ΔPMAsΔmel was constructed by two transformations in succession and screened for both NAT and HPT resistance.

### 2.7 Identification of polymalic acid content

The engineered strain *A. pullulans* BL06 was cultivated in a pullulan producing medium at 28°C and 200 rpm for 120 h. After centrifugation at 14,000 × g for 10 min, 1 mL of fermentation supernatant was mixed with 1 mL of sulfuric acid solution (1 M) and placed at 90°C for 12 h. The reaction supernatant was centrifuged at 14,000 × g for 10 min to be filtrated with 0.22 μm syringe filters before HPLC analysis. The Bio-Rad Aminex HPX-87H column was used to determine the concentration of polymalic acid under an ultraviolet detector at 210 nm. The mobile phase was 5 mM sulfuric acid solution with a flow rate of 0.6 mL/min at 65°C.

### 2.8 Fermentation process of *A. pullulans* strains for pullulan production in a 5 L bioreactor

The pullulan production of *A. pullulans* BL06 and *A. pullulans* BL06ΔPMAs were conducted *via* batch fermentation in a 5 L bioreactor for 120 h. The preserved strains were streaked on YPD plates and cultured at 28°C for 48–72 h. The single colonies were picked up and inoculated in 5 mL YPD medium. After cultivation at 28°C and 200 rpm for 20 h, the culture broth was transferred into 300 mL fresh YPD medium in a shake flask for another 16 h cultivation at the same condition. The obtained 300 mL of seed culture was then added into a 5 L bioreactor at 10% (V/V) of the dose. The total liquid content was 60% (V/V) of the bioreactor The cultivation was performed at 28 ± 2°C with a stirring speed of 500 rpm. Once the dissolved oxygen was reduced to 20%, the stirring speed was increased to 800 rpm and the ventilation was maintained at 3 vvm. After 36 h fermentation, 800 mL of sucrose liquid (140 g/L) was feeding at 100 mL/h. During the fermentation, 50 mL of the culture was harvested at 12 h interval for product detecting. The fermentation medium components in the bioreactor included 11.6% (w/v) sucrose, 0.28% (w/v) yeast extract powder, 0.07% (w/v) ammonium sulfate, 0.45% (w/v) K_2_HPO_4_, 0.02% (w/v) MgSO_4_·7H_2_O, 0.09% (w/v) NaCl.

### 2.9 Application of the novel moderate Mw pullulan in food preservation

The solution (30 g/L) of purified pullulan produced by *A. pullulans* BL06 ΔPMAs was spread evenly to the surface of celery cabbages and mangos, respectively. The original weights of the selected samples were similar. After laying at room temperature (25°C–35°C) with a relative humidity of 68%–75% for different times (1 day, 2 days, 3 days, to 15 days), the surface state of the samples was observed and the weight loss of the samples was determined, respectively. The samples with no pullulan spread were used as controls.

### 2.10 Analytic methods and data availability

The statistical analysis was carried out using one-way analysis of variance followed by Duncan’s multiple comparison tests. *p* values < 0.05 were considered statistically significant. The results are presented as the mean ± the standard deviation (SD) for a replication of *n* = 3. The ITS sequence of *A. pullulans* BL06 was deposited in the GenBank database with the accession number OP810667. The gene sequences of AGSII, mel, and PMAs were based on the records in the GenBank database with accession numbers MH917125.1, KT429644, and MN551082, respectively.

## 3 Results and discussion

### 3.1 A high-yield strain of high-Mw pullulan was screened from the environment

Through morphologically screening of the colonies on the PDA plate, 16 strains forming with smooth, moist, and yeast-like properties, as shown in Figure 1C, were chosen for further evaluation of their ability to produce extracellular polysaccharides (EPS). Each of the strains was incubated in a pullulan producing media at 28°C and 200 rpm for 7 days. As shown in Figure 1A, all 16 strains showed the ability to produce EPS. Among those, the strains BL06, BL13 and BL17 produced the relatively highest EPS amount (≥37 g/L). At the same time, the rheological behavior of the extracellular polysaccharides produced by the 16 strains was determined. As the results showed in Figure 1B, the viscosity of the EPS produced by most strains was lower than 1,000 mPa·s, and only strain BL06 showed the significantly highest viscosity of 1,912 mPa·s at 37.9 g/L EPS. However, similar amounts (39 g/L and 38.3 g/L) of EPS production of strains BL13 and BL17 showed viscosities of only 163 mPa·s and 174 mPa·s, respectively (Figures 1A, B). Thus, the remarkably higher viscosity makes us interested in further identifying the properties of the EPS produced by strain BL06. Before identifying the EPS properties of strain BL06, the ITS sequence of strain BL06 was determined and uploaded to the GenBank database. After the search for similarities between ITSs of the isolate strain BL06 and those of the type strains in the National Center for Biotechnology Information (NCBI) database, a phylogenetic tree of ITSs that showed higher than 98% identity with that of strain BL06 was constructed, as shown in Figure 1D. The results showed that many phylogenetically related *Aureobasidium spp*. were similar to strain BL06 and the most closely related strain was *A. pullulans* (Figure 1D). So the newly isolated strain BL06 was identified as *A. pullulans* BL06 in this study. According to previous reports, *Aureobasidium pullulans* species are known to be hosts for high level production of pullulan (Ma et al., 2014; Liu et al., 2021).

### 3.2 Production properties of the yeast-like fungus Aureobasidium pullulans BL06

In order to further identify the high viscosity EPS produced by *A. pullulans* BL06. The EPS in 3 L of fermentation broth was purified by the alcohol precipitation method to obtain 90 g white dry powders (Figure 2A). The purified EPS was analyzed by FT-IR. As shown in Figure 2B, in comparison with the commercial pullulan standard as a control, except for a peak at 1730 cm^−1^, all of the characteristic peaks of EPS from strain BL06 were consistent with pullulan. The intense absorbance peak appears at 3,325 cm^−1^, which was the unique characteristic of the presence of repeating units of OH groups in sugars. The peak at 2,918 cm^−1^ showed the presence of C-H stretching. The absorption at 842 cm^−1^ was attributed to the presence of α-D-glucopyranoside bonds whereas a band at 752 cm^−1^ revealed the presence of α-(1, 4)-D-glucosidic bonds, and a strong absorption at 993 cm^−1^ was characteristic of α-(1, 6)-D-glucosidic bonds. The strong peaks at approximately 1640 cm^−1^ and 1360 cm^−1^ were characteristic of glycosidic linkage O-C-O bonds and C-O-H bending, respectively. These absorption patterns at various stretching frequencies confirmed that the purified EPS produced by *A. pullulans* BL06 had all the pullulan-like peaks and was identified as pullulan. The Mw of the purified pullulan was 3.3 × 10^6^ Da determined by the HPGPC-MALLS method (Supplementary Figure S2). It is significantly higher than that of the previously reported level (Jiang et al., 2019; Singh et al., 2019). This result could also explain why the exopolysaccharides produced by strain 06 showed remarkably higher viscosity at similar EPS concentration compared with other strains (Figures 1A, B). Because pullulan as a straight chain polymer, the property of viscosity was mainly affected by its Mw and concentration (Singh et al., 2021). Thus, the ability to produce high-Mw pullulan endowed *A. pullulans* BL06 with important value in scientific research and industrial applications.

To determine the purity of extracellular pullulan produced by *A. pullulans* BL06, the pullulan synthase (AGSII) gene (Figure 2D) was knocked out from its genome. Through weighting the purified extracellular alcohol precipitation products (Figure 2A) of *A. pullulans* BL06 ΔAGSII, the pullulan purity produced by *A. pullulans* BL06 was calculated to be no less than 96.9%. Besides, based on the specific peak at 1730 cm^−1^ of samples produced by *A. pullulans* BL06 revealing the presence of -C = O (Figure 2B) and *A. pullulans* strains were also reported to yield a high level of polymalic acids (Figure 2D) (Zeng et al., 2020), the impurities were estimated as polymalic acids. To further prove the estimation, the alcohol precipitation products of *A. pullulans* BL06 (Figure 2A) were identified by HPLC after acid hydrolysis. The results showed that the peak of the acid hydrolysis products was consistent with the characteristic peak of the L-malic acid standard (Figure 2C). Though quantitative calculation according to the methods described in Section 2.7, *A. pullulans* BL06 screened in this study produced a proportion (3%) of polymalic acids during the production of the high-Mw pullulan.

### 3.3 Strain modification of A. pullulans BL06 for the production of pullulan

**FIGURE 4:**
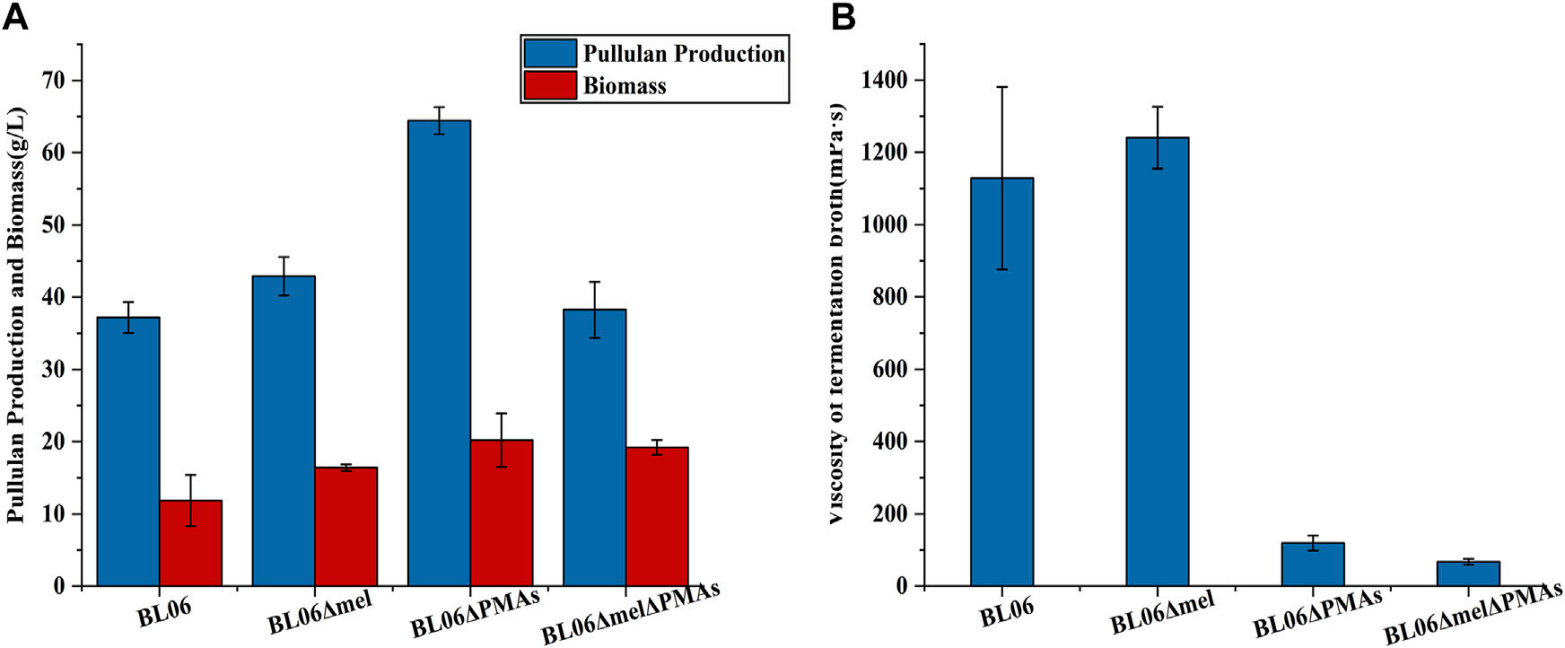
Pullulan production and viscosity of fermentation broth of the engineered strains. **(A)** Pullulan production and biomass of the engineered strains after 120 h fermentation; **(B)** Viscosity of fermentation broth of the engineered strains after 120 h fermentation. All values are expressed as the means ± SDs (*n* = 3).

The byproducts (polymalic acid) produced by *A. pullulans* BL06 will affect the purification of pullulan (Figure 2A) from the fermentation broth to obstruct its application in various fields. Besides, the synthesis of polymalic acid also competed for the substrate glucose-6-phosphate (Figure 2D) to reduce the amount of pullulan in *A. pullulans* BL06. Thus, an engineered strain *A. pullulans* BL06ΔPMAs was constructed through knocking out the encoding gene for the polymalic acid synthase (PMAs) in the genome of *A. pullulans* BL06. Moreover, according to previous reports, *A. pullulans* species strains usually synthesize melanin, thereby being named “black yeast” (Liu et al., 2020). While the melanin production is well known as an obstacle to pullulan industrial production because it increases the cost of pullulan purification (Liu et al., 2020). Therefore, a melanin deficiency strain *A. pullulans* BL06Δmel and a double gene knockout strain *A. pullulans* BL06ΔmelΔPMAs were also constructed in this study. All three engineered strains were verified to have the target genes knocked out correctly (Figures 3C–E). Compare with the titer of pullulan produced by wild-type *A. pullulans* BL06, all three engineered strains showed increase in the titer of pullulan (Figure 4A). In particular, *A. pullulans* BL06ΔPMAs produced pullulan at the highest yield level of 64.43 g/L after 120 h of fermentation, which was 1.7 times higher than that of *A. pullulans* BL06 (Figure 4A). *A. pullulans* BL06Δmel and *A. pullulans* BL06ΔmelΔPMAs produced slightly higher amounts of pullulan than *A. pullulans* BL06, while taking the growth enhancement of the two strains into account indicated that melanin deficiency could fail to increase pullulan production and even reduce it (Figure 4A). As a previous report, the pullulan biosynthetic genes *upt*, *pgm*, *ugp*, and *pul* were downregulated, while the negative regulatory gene (*creA*) of pullulan synthesis was upregulated by melanin deficiency (Liu et al., 2021). In this study, the melanin deficiency regulation effect was more significant in *A. pullulans* BL06ΔPMAs (Figure 4A). Besides, melanin deficiency did not affect the viscosity of extracellular pullulan products, while the viscosity of pullulan (119 mPa·s) was decreased remarkably due to knocking out PMAs. Based on determining the Mw (1.3 × 10^5^) of the extracellular pullulan produced by *A. pullulans* BL06ΔPMAs (Supplementary Figure S3), the decrease in viscosity was considered as be caused by the reduction of Mw of pullulan. Thus, a novel engineered strain, *A. pullulans* BL06ΔPMAs, showed a high level of extracellular moderate Mw pullulan was realized in this study. Through the results, we also conclude that regulating the expression of PMAs in *A. pullulans* BL06 may effectively change the Mw and titer of pullulan in *A. pullulans* BL06. The specific regulatory mechanism needs to be further revealed.

### 3.4 The scale-up fermentation of the pullulan production strains in a 5 L bioreactor

**FIGURE 5:**
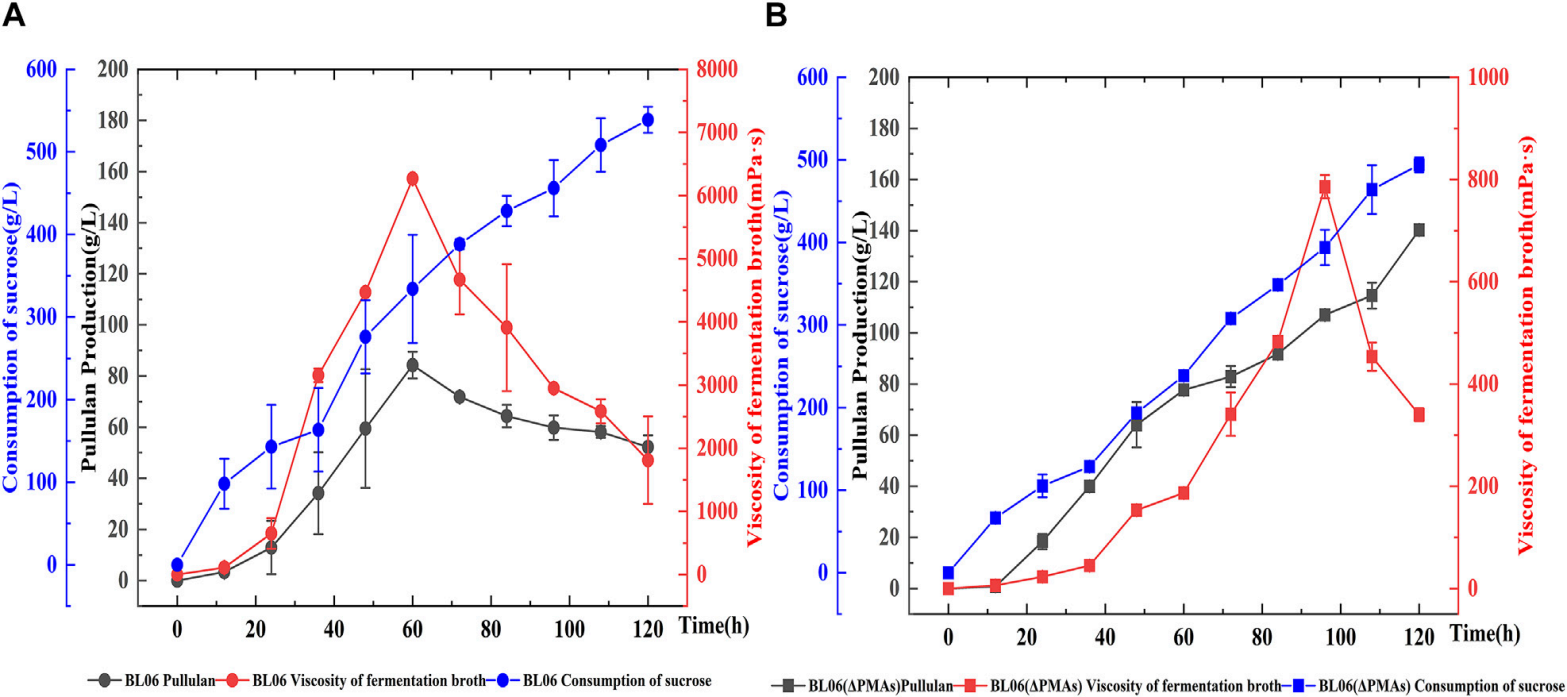
Batch feeding fermentation of *A. pullulans* BL06 **(A)** and *A. pullulans* BL06ΔPMAs **(B)** in a 5 L bioreactor. The black solid circle and solid square indicate the pullulan production by *A. pullulans* BL06 and *A. pullulans* BL06ΔPMAs respectively; the red solid circle and solid square indicate the viscosity of the fermentation broth of *A. pullulans* BL06 and *A. pullulans* BL06ΔPMAs respectively; the blue solid circle and solid square indicate the consumption of sucrose by *A. pullulans* BL06 and *A. pullulans* BL06ΔPMAs respectively. All values are expressed as the means ± SDs (*n* = 3).

A batch feeding fermentation of *A. pullulans* BL06 and *A. pullulans* BL06ΔPMAs in a 5 L bioreactor was performed to produce high Mw pullulan and moderate Mw pullulan, respectively. The highest yield (84.3 g/L) of extracellular high-Mw pullulan with a high viscosity of 6,270 mPa·s was obtained for *A. pullulans* BL06 at 60 h of fermentation (Figure 5). After 60 h, the high-Mw pullulan was degraded gradually associating the viscosity decreases (Figure 5). However, the yield of the moderate-Mw pullulan produced by *A. pullulans* BL06ΔPMAs gradually increased during fermentation. At 120 h of fermentation, the highest yield of the moderate-Mw pullulan was up to 140.2 g/L with a viscosity of 340 mPa·s (Figure 5). It is the highest level of pullulan to date. Besides, the sucrose consumption profiles of the two strains were similar, while *A. pullulans* BL06ΔPMAs consumed relatively less sucrose substrate (Figure 5). Thus, the novel engineered strain *A. pullulans* BL06ΔPMAs could vastly reduce the production cost and expand the application scope and potential of pullulan.

### 3.5 The application effect of novel pullulan in food preservation

**FIGURE 6:**
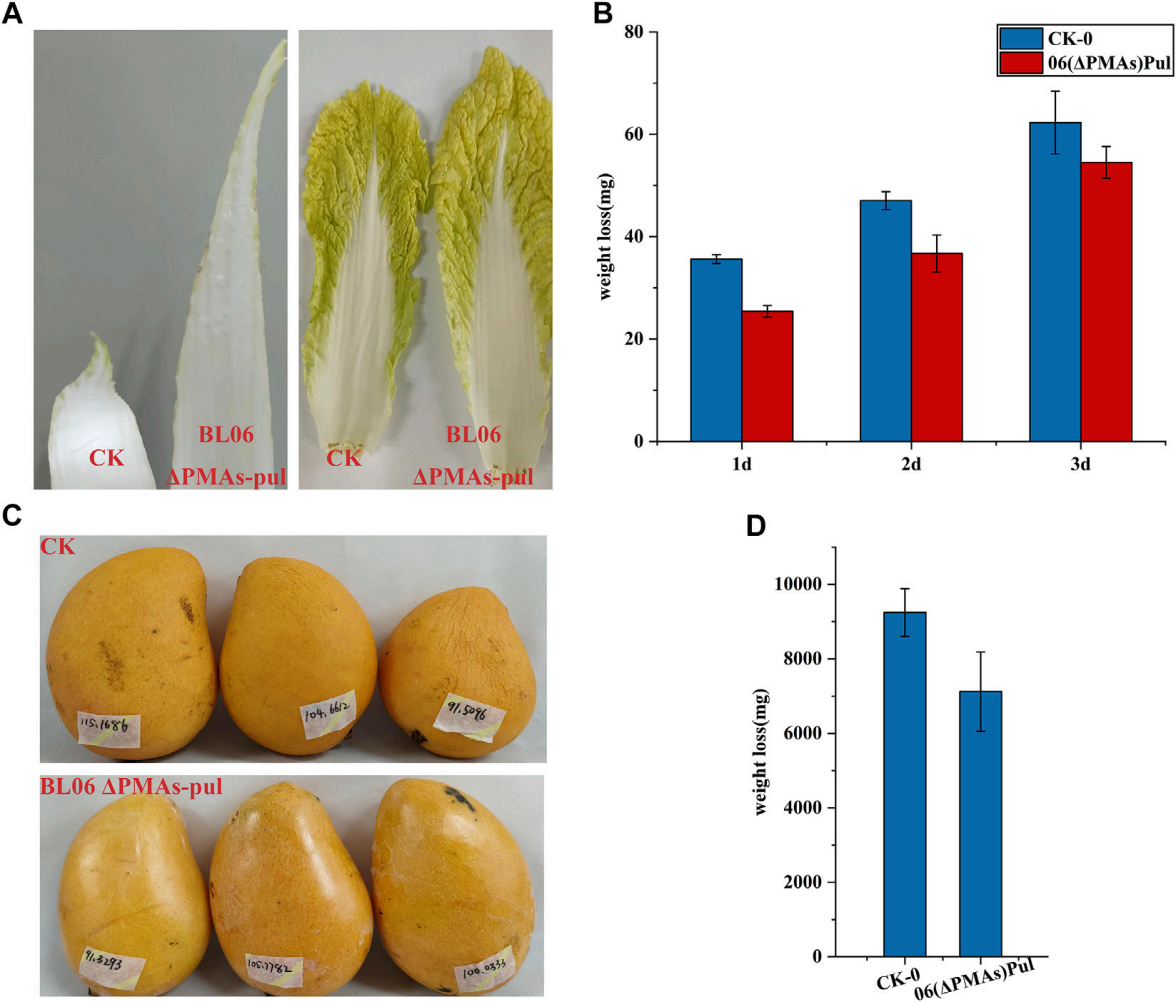
Food coating application of the moderate Mw pullulan films. **(A)** Comparison of apparent states of celery cabbages smeared with pullulan films or not; **(B)** Comparison of apparent states of mangos smeared with pullulan films or not; **(C)** Comparison of weight loss of celery cabbages smeared with pullulan films or not; **(D)** Comparison of weight loss of mangos smeared with pullulan films or not; All values are expressed as the means ± SDs (*n* = 3).

Pullulan is an edible polymer and has been certified to be harmless for usage in food products by food safety regulations in many countries (Singh et al., 2019). Pullulan solutions can form clear films or coatings that are oxygen impermeable, oil resistant and have good mechanical properties (Singh et al., 2019). Owing to their properties, pullulan films have the potential to be used as versatile and novel packaging materials. Combined with the high production level of the moderate-Mw pullulan obtained in this study will significantly reduce its application cost. Thus, the moderate-Mw pullulan produced by *A. pullulans* BL06ΔPMAs was used as a protective film smearing evenly to the surface of celery cabbages and mangos, respectively. After 3 days and 15 days of laying at room temperature environment (25–35°C and 68%–75% humidity), the celery cabbages and mangos coated with pullulan remained fresh and had a good appearance, while the control group was shrunken and wrinkled (Figures 6A, B). Further weighting of the samples showed that the pullulan coating reduced the weight loss of celery cabbages and mangos by 12.5% and 22.0%, respectively, compared with the controls (Figure 6C, D). The results indicated that the moderate-Mw pullulan developed in this study could be used as a food coating to enhance the shelf-life of vegetables and fruits, which protects them from dehydration and spoilage.

## 4 Conclusion

In this study, a novel strain *A. pullulans* BL06 with a high production level for high Mw pullulan was firstly screened and identified. By regulating the key synthesis pathway of pullulan impurities, we found that knocking out the key genes not only affected the yield and purity of extracellular pullulan but also affected the Mw of pullulan products. The engineered strain *A. pullulans* BL06ΔPMAs with the highest enhancement in pullulan production was discovered to produce the extracellular pullulan with a mean moderate Mw. After scale-up fermentation in a 5 L bioreactor, the highest production of the high-Mw (3.3 × 10^6^ Da) pullulan was 84.3 g/L with a viscosity of 6,270 mPa·s produced by *A. pullulans* BL06, and the highest production of the moderate-Mw (1.3 × 10^5^ Da) pullulan was up to 140.2 g/L with a viscosity of 340 mPa·s produced by *A. pullulans* BL06ΔPMAs, respectively. Through the two strains and the key regulated genes obtained in this study, pullulan products with various Mw ranges will be synthesized efficiently in further work. Besides, the high production level of the moderate-Mw pullulan obtained in this study was certified for use as a food coating to enhance the shelf-life of vegetables and fruits. However, the application of the various pullulans developed in this study is definitely not limited to food preservation.

## Data Availability

The original contributions presented in the study are included in the article/Supplementary Material, further inquiries can be directed to the corresponding author.
