# Supplementary material for: High-level production of pullulan and its biosynthesis regulation in Aureobasidium pullulans BL06
Source: Front Bioeng Biotechnol. 2023 Jan 26;11:1131875. doi: 10.3389/fbioe.2023.1131875 (PMC9909216; doi:10.3389/fbioe.2023.1131875)
Supplement: Supplementary file 1 [file Table1.DOCX]

Supplementary Material

High-level production of pullulan and its biosynthesis regulation in *Aureobasidium pullulans* BL06

Shuyu Chen, Hongchen Zheng, Jiaqi Gao, Hui Song, Wenqin Bai*

*** Correspondence:** Wenqin Bai: [baiwq@tib.cas.cn](mailto:baiwq@tib.cas.cn)

**Table S1. Plasmids used in this study.**

| Plasmids | Properties |
| --- | --- |
| pJQ046 | ΔPMAs 5arm-P_tef_-hpt-T_tef_-3arm |
| pSY005 | Δmel 3arm-P_pgk_-nat-T_poly_A-5arm |
| pSY018 | ΔAGSⅡ 3arm-P_pgk_-nat-T_poly_A-5arm |

**Table S2. Primers used in this study.**

| Primer Name | Primer Sequence |
| --- | --- |
| ITS1 | TCCGTAGGTGAACCTGCGG |
| ITS4 | TCCTCCGCTTATTGATATGC |
| backbone-F(PUC57) | GGTCTCCTGCAGcctagggcgttcggc |
| backbone-R(PUC57) | GGTCTCaCCaggtggcacttttcggg |
| Δmel-5F | GGTCTCcctGGTCCTCCCACCACTACTAAGCG |
| Δmel-5R | ggtctCGAATTcACGATGGCAATCTTGTCGTTCTGAG |
| Δmel-3F | GGTCTCggatccCTGGTACCCAGGCTGGTGAC |
| Δmel-3R | GGTCTCCTGCAGCTGGGTTCTCAGAAAGGTAGCCC |
| ΔPMAs-5F | GGTCTCcctGGTTTCGTCATCGAGTCCTGGGGATTG |
| ΔPMAs-5R | GGTCTCGAATTCGAAGTGTCCAGTACGTCGTTCTC |
| ΔPMAs-3F | GGTCTCGGATCCCTCTGGCGGACCGCCAC |
| ΔPMAs-3R | GGTCTCCTGCAAAGAGTAAATCGATGGGCAAGC |
| ΔAGSⅡ-5F | ggtctccctggtAACCAAACGGAGGCACAACTC |
| ΔAGSⅡ-5R | ggtctcCAGGCTGTGCGCTGGGTTTCT |
| ΔAGSⅡ-3F | ggtctcAGCCTGGTCTCGGTGCCCCA |
| ΔAGSⅡ-3R | ggtctcctgcaATCTATGTCTGAATTCTATAAACTACTACGTCAAAG |
| Res-F | ggtctcgaattcgtcgacaacccttaatTACCGt |
| Res-R | GGTCTCggatccggatctgatatcacctaTACCGttcG |


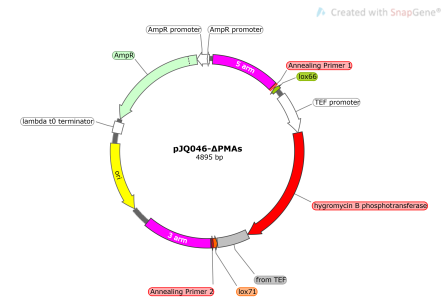

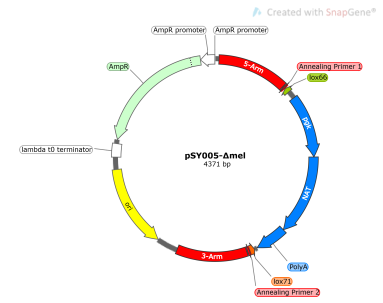

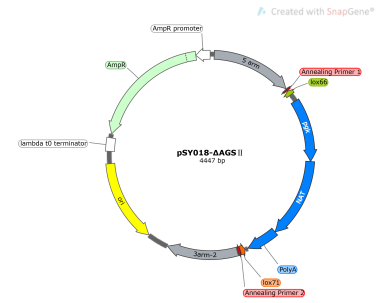


**A**

**C**

**B**

**Supplementary Figure S1.** The recombinant plasmids for knocking out the gene of PMAs (A), mel (B) and AGSII (C) from the genome of *Aureobasidium pullulans* BL06.


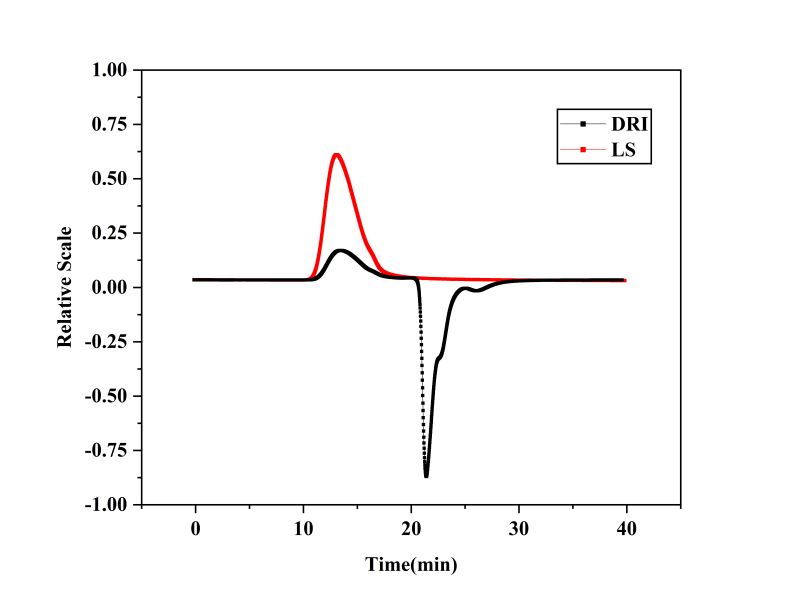


**Supplementary Figure S2.** Molecular weight determination of *A. pullulans* BL06 pullulan by HPGPC-MALLS.


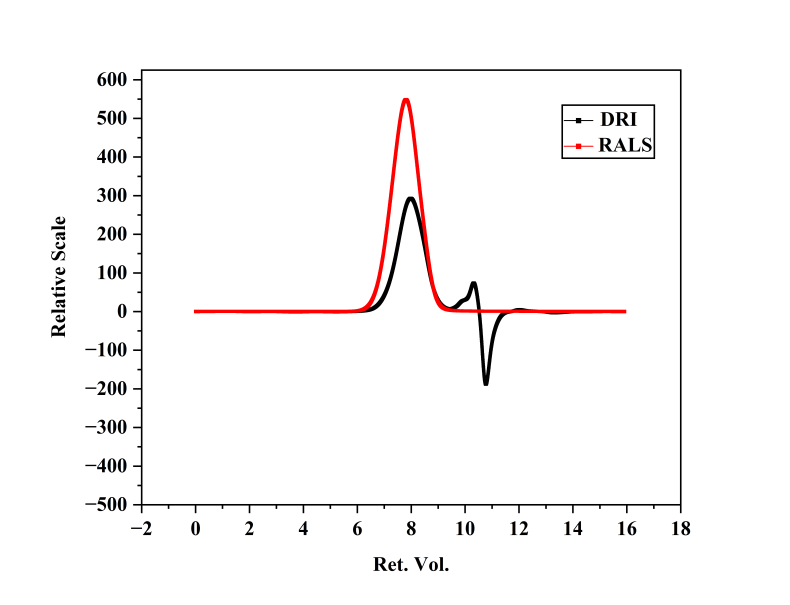


**Supplementary Figure S3.** Molecular weight determination of *A. pullulans* BL06 (ΔPMAs) pullulan by RALS.
